# *In vitro* investigations of a novel wound dressing concept based on biodegradable polyurethane

**DOI:** 10.1088/1468-6996/16/3/034606

**Published:** 2015-05-20

**Authors:** Markus Rottmar, Michael Richter, Xenia Mäder, Kathrin Grieder, Katja Nuss, Agnieszka Karol, Brigitte von Rechenberg, Erika Zimmermann, Stephan Buser, Andreas Dobmann, Jessica Blume, Arie Bruinink

**Affiliations:** 1 Laboratory for Biointerfaces, Empa, Swiss Federal Laboratories for Materials Science and Technology, Lerchenfeldstr. 5, CH-9014 St. Gallen, Switzerland; 2 MSRU Vetsuisse Faculty ZH, University of Zurich, Winterthurerstr. 260, CH-8057 Zurich, Switzerland; 3 CABMM, University of Zurich, Winterthurerstr. 260, CH-8057 Zurich, Switzerland; 4 nolax AG, Eichenstr. 12, CH-6203 Sempach Station, Switzerland

**Keywords:** polyurethane, wound treatment scaffold, cell culture, biocompatibility, degradation

## Abstract

Non-healing and partially healing wounds are an important problem not only for the patient but also for the public health care system. Current treatment solutions are far from optimal regarding the chosen material properties as well as price and source. Biodegradable polyurethane (PUR) scaffolds have shown great promise for *in vivo* tissue engineering approaches, but accomplishment of the goal of scaffold degradation and new tissue formation developing in parallel has not been observed so far in skin wound repair. In this study, the mechanical properties and degradation behavior as well as the biocompatibility of a low-cost synthetic, pathogen-free, biocompatible and biodegradable extracellular matrix mimicking a PUR scaffold was evaluated *in vitro*. The novel PUR scaffolds were found to meet all the requirements for optimal scaffolds and wound dressings. These three-dimensional scaffolds are soft, highly porous, and form-stable and can be easily cut into any shape desired. All the material formulations investigated were found to be nontoxic. One formulation was able to be defined that supported both good fibroblast cell attachment and cell proliferation to colonize the scaffold. Tunable biodegradation velocity of the materials could be observed, and the results additionally indicated that calcium plays a crucial role in PUR degradation. Our results suggest that the PUR materials evaluated in this study are promising candidates for next-generation wound treatment systems and support the concept of using foam scaffolds for improved *in vivo* tissue engineering and regeneration.

## 1. Introduction

Badly healing and non-healing wounds are problematic not only for the individual patient but also for society at large because extensive and repeated treatment inflicts very high costs on the public health care system [1]. Therefore, not only is prompt closure of skin wounds of great importance for good patient care, but the demand for inexpensive and effective treatments for badly healing and non-healing wounds is of enormous curative, social, and industrial interest.

Skin wounds can have a different etiology, including physical (e.g., prolonged compression resulting in bedsores) or thermal injury as well as the presence of underlying medical or physiological conditions (e.g., diabetes). Accordingly, they can be roughly classified into acute and chronic wounds. Whereas acute wounds usually heal completely within a reasonable time frame of up to six weeks, chronic wounds take very long to heal and often never really close or re-occur [2]. Different chronic wounds have individual characteristics. The prognosis for complete closure depends on a patient’s health status, wound type (i.e., venous versus non-venous), wound size, vascularization, microbiological status, and, especially with diabetic wounds, patient compliance.

Generally, the healing of acute wounds encompasses the phases of inflammation, proliferation (encompassing granulation and epithelialization), and remodeling/maturation, with the latter phases being dominated by the formation and remodeling of the extracellular matrix (ECM). When any one of the steps in this process is compromised, wound healing can stagnate, which usually results in ECM degradation [3–5]. The ECM is, however of crucial importance for tissue regeneration because it serves as a matrix for the ingrowth of cells into the wound [6].

Commonly used wound care dressing materials include films, hydrogels, hydrofibers, and foams, with each class having distinct advantages and disadvantages [7]. However, current wound treatment concepts as well as products are based mainly on the application of natural or artificial scaffolds to mimic the structural environment of the intact human dermis [8, 9]. These scaffolds include but are not limited to a range of dermal matrices [10] and alginate dressings [11] as well as materials and composites such as chitosan/PLA [12], PVA/alginate [13], cellulose/collagen [14], and silk [15]. Despite the progress that has been made with artificial dressings, most of the commercially available cell adhesion–promoting scaffolds contain collagen [9], which has the potential to transmit pathogens or to elicit hypersensitivity [16]. In addition, wound care based on the application of such scaffolds is usually very expensive [7]. Due to their good biocompatibility, polyurethanes (PURs) have been of great interest in the medical field in recent years; this has ultimately led to the introduction of biodegradable PU scaffolds [17–21]. However, a slow degradation velocity (>several weeks to months for complete degradation) of these scaffolds limits their potential use for the treatment of skin wounds. The mechanisms for increased degradation are rather unknown [22], and therefore, the goal of parallel scaffold degradation and new tissue formation has not been able to be achieved to date.

To design a novel wound dressing that matches the challenging criteria of a degradable scaffold for *in vivo* tissue engineering, many issues need to be solved. First and foremost, the raw materials need to be inexpensive and available from various sources, and they must be biocompatible and biodegradable according to International Organisation for Standardisation (ISO) standards 10993-5 and 10993-13. In the case of a polyurethane scaffold, polyurethane being a heterogenic polymer, this is especially difficult because all ingredients, including the monomers, pre-polymers, organic and metal–organic catalysts, and reaction products, must meet these requirements. From an analytical point of view, in contrast with some predictable leachables, the *in vitro* degradation and identification of toxicologically relevant degradation products of a polyurethane matrix under various conditions cannot be easily monitored, identified, or even fully covered by standard chemical methods. An additional drawback is that the currently used ISO standard 10993-13 falls short in terms of development of meaningful degradation studies.

**Figure 1.**
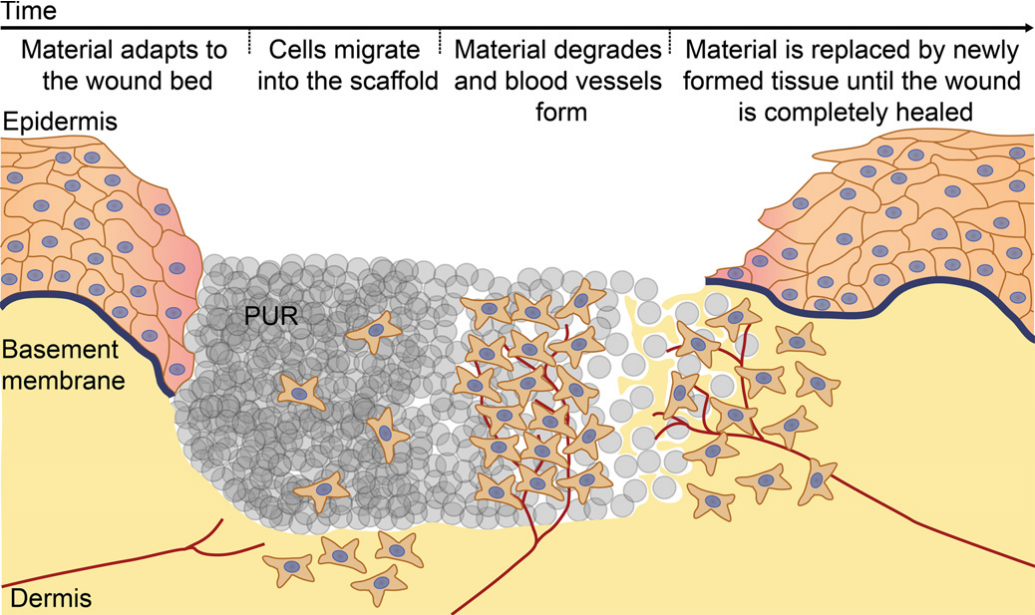
The *in vivo* tissue engineering based wound treatment concept. The series of events envisioned during wound repair supported by the biodegradable PUR scaffold. Upon wetting, the PUR scaffold adapts to the wound bed, allowing cells to easily migrate into the scaffold. The scaffold is degraded and blood vessels form until the material is completely replaced by newly formed tissue, eventually leading to a healed wound.

The aim of this study was to evaluate an *in vivo* tissue engineering wound treatment concept based on a completely synthetic biodegradable PUR scaffold that is expected to serve as an artificial non-cytotoxic ECM to support wound healing by facilitating cell adhesion and ingrowth. A second aim was to define one or more factors affecting the biodegradation of the scaffold in the wound in order to be able to steer its degradation rate by modification of the production process and the ratios between the various components. Figure 1 depicts the series of events envisioned during wound repair supported by the new biodegradable PUR scaffold. Characterization of the PUR scaffolds showed that the overall pore structure is independent, whereas the degradation rate and cell adhesion are greatly dependent, on the exact formulation of the material. Furthermore, it was found that calcium ions were affecting the degradation rate. The evaluated set of materials was found to be, irrespective of the formulation, not toxic; and in evaluating the adhesion of single cells as well as the outgrowth from clustered cell spheroids, a PUR formulation could be identified that shows good cell adhesion and cell ingrowth. In conclusion, the studied PUR scaffolds showed great promise for supporting wound healing, thus warranting additional *in vivo* studies.

## 2. Materials and methods

### 2.1. Characterization of PUR scaffolds

For qualitative evaluation of the scaffold structure, scanning electron microscopy (SEM) was employed. For this, a 10 nm-thick layer of gold was applied (Polaron Equipment, SEM Coating Unit E5100, Kontron AG, Switzerland; 5 mA, 1 mbar, 10 min; 10 nm-thick coating) on the PUR scaffolds, and the samples were imaged at 30x magnification with a Hitachi S-4800 scanning electron microscope (Hitachi High-Technologies, Canada) at an accelerating voltage of 10 kV and with 10 *μ*A current flow.

For quantitative evaluation of different structural indices of the PUR scaffolds, a high-resolution microcomputed x-ray system (*μ*CT 40, Scanco Medical, Brüttisellen, Switzerland) without the addition of a contrasting agent was chosen. The specimens were scanned at an energy of 45 kV and an intensity of 176 *μ*A. An integration time of 300 ms and two-times averaging were used to enhance the signal-to-noise ratio because of the low adsorption coefficient of the PUR. Two-dimensional CT images were scanned and reconstructed in 2048 × 2048 pixel matrices from 1,000 projections using a standard convolution–backprojection procedure. Images were stored in 3D arrays with an isotropic voxel size of 6 *μ*m. After the images were pre-processed with the same Gaussian filter to partially suppress the noise, the gray-value images were segmented with different threshold values to extract the foam or the aggregate matrix. Structural indices of the foams were computed and determined using a computer analysis method. The structural parameters reported are porosity (%), average macropore size (*μ*m), and average wall thickness (*μ*m).

### 2.2. Degradability and mechanical properties of PUR scaffolds

The degradation experiments were carried out according to ISO 10993-13:2009-08. The specimens (3 × 3 × 0.5 cm^3^) were incubated in H_2_O_2_ at 37 °C (1 g: 10 ml) to mimic the oxidative degradation occurring in the wound. The peroxide solution was changed every 3–4 days to maintain a constant degradation milieu. The specimens were dried in a desiccator over P_2_O_5_ until reaching weight constancy before and after degradation assay to determine weight loss. The specimens were separated from the peroxide solution by centrifugation using a PALL Macrosep Advance 0.2 *μ*m Supor Membrane at 3,900 rpm.

Mechanical tests were performed as described previously [23], with the following modifications: compression tests with 2 × 3 × 4 cm blocks and elasticity tests with 2 × 1 × 0.8 cm membranes in triplicate with 5% strain/min on an Instron 5567 setup from Instron Corporation, UK.

### 2.3. High-performance liquid chromatography–mass spectrometry

For the high-performance liquid chromatography–mass spectrometry (HPLC-MS), the foam was incubated in water or culture media overnight at 37 °C. The supernatants were then analyzed using an RP18 column as the stationary phase (Phenomenex Gemini 250 × 2 mm; 5 *μ*m) on an Agilent 1100 series system with a diode array detector coupled to an esquire HCT ion trap mass spectrometer (Bruker, Germany) equipped with an electrospray ionization (ESI) source and operated in the positive mode. An isocratic elution was carried out at a flow rate of 0.5 ml min^−1^ using acetonitrile/water 10% (v/v) containing 0.1% (v/v) formic acid; the 2,2-dimorpholinodiethylether (DMDEE) was determined to be *m/z* 244.9 [M+H]^+^ (calculated exact mass 244.18 g mol^−1^; extracted ion current chromatogram mass processed with HyStar). The mass spectra were verified by using the DMDEE raw material as the external and internal standard. The important ionization parameters were chosen as follows: dry temperature: 360 °C, HV capillary: 4600 V, nebulizer: 40 psi, dry gas: 8.0 l min.^−1^ ICP-OES measurements of the aqueous foam extracts revealed a concentration of Bi ions lower than 0.5 ppm (using an ICP-OES Optima 3000, Perkin Elmer). Thus, the foam samples were treated according to a standard procedure [23] with nitric acid plus sonication and were subsequently diluted in water to a defined volume. Optical analysis was carried out at the emission of Bi at 223.061 and 306.766 nm.

### 2.4. Material extracts and pre-incubated PUR scaffolds

Material extracts were prepared according ISO10993-5/12. Briefly, PUR scaffolds were cut into discs of 20 mm and placed in six-well plates and 2.1 ml culture medium (Dulbecco’s modified eagle medium (DMEM) supplemented with 10% fetal calf serum (FCS); 2 mM of L-glutamine and 5% penicillin/streptomycin (pen/strep)) were added. Following 24 h incubation at 37 °C with 5% CO_2_, the extracts were used for 3-(4,5-dimethylthiazol-2-yl)-2,5-diphenyltetrazolium bromide (MTT) and protein assays and HPLC-MS (culture medium without FCS and pen/strep), whereas the PUR scaffolds were used for cell adhesion and colonization assays.

### 2.5. MTT and protein assays

The effect of material extracts on cellular activity was assessed by an MTT assay as described previously [24]. In brief, 24 h after seeding at a density of 10 000 cells per 96-well plate well, 3T3 fibroblast cultures (ECACC 85022108, Sigma) were treated for 24 h by a control medium or material extracts. Subsequently, the cells were washed once with plain DMEM before the addition of 25 *μ*l of an MTT solution (5 mg ml^−1^) to each well for 1 h at 37 °C. The solution was then removed, and intracellular MTT–formazan crystals were dissolved in 90% (v/v) ethanol for 10 min. Absorbance was measured at 550 nm, and wells without cells were used as blanks. For measurement of the influence of material extracts on total protein content as an index of total cell mass, a bicinchoninic acid (BCA) protein assay (Pierce Nr. 23225; Thermo Fisher Scientific, USA) was employed. In brief, the medium was removed and the cells were washed twice with phosphate buffered saline (PBS, pH 7.4) prior to the addition of 200 *μ*l of BCA solution (1:50 reagent A:B) in a rotary shaker for 1 h at room temperature (RT). Absorbance was measured at 550 nm, wells without cells were used as blanks, and serial dilutions of bovine serum albumin were used as the standard.

### 2.6. Fibroblast cell adhesion on and colonization of PUR scaffolds

Pre-incubated PUR scaffolds were placed in six-well plates, and 2.1 ml of culture medium was added. Sterilized 10 mm glass rings were placed on top to keep the scaffolds submersed, and 3 × 10^5^ mouse 3T3 fibroblasts were seeded on top of the scaffolds. Alternatively, cell spheroids consisting of 1.5 × 10^4^ human dermal fibroblasts (HDFs, expanded in the laboratory from a biopsy of adult abdominal skin as described previously [25]) were formed by centrifugation for 5 min at 200 rpm and subsequent culture in polypropylene tubes for 24 h before the spheroids were transferred to on top of the PUR scaffolds. The culture medium was changed after 24 h and every 2–3 days thereafter. On indicated days, the cells were fixed with 4% paraformaldehyde (PFA)/0.2% Triton X-100 (TX-100) for 8 min and stored in PBS until staining.

### 2.7. Immunohistochemistry

For immunohistochemical staining of cells cultivated on the PUR scaffolds, antibodies/dyes were diluted in PBS and incubation steps were performed at RT for 1 h. Actin and the nuclei were stained using Alexa546 conjugated phalloidin (1:40, Molecular Probes, B607) and DAPI (4,6-diamidino-2-phenylindole, 1:1000, Sigma–Aldrich, D9542), respectively. Samples were washed extensively with PBS before imaging on a confocal laser scanning microscope (LSM780, Zeiss, Oberkochen, Germany). Due to the 3D nature of the scaffolds, the images were acquired in z-stacks and presented as maximum-intensity projections.

### 2.8. Live/dead viability assay

Mouse fibroblasts (3T3) cultivated for 1 and 4 days on PUR scaffolds were evaluated with a live/dead viability kit (Invitrogen, L3224) according to the manufacturer’s instructions with minor modifications. In brief, the culture medium was removed and the cells were cultivated in a fresh medium supplemented with 1.3 *μ*M ethidium homodimer-1 and 2.6 *μ*M calcein AM for 10 min at 37 °C. Subsequently, the cells were washed with fresh culture medium and imaged on a confocal laser scanning microscope equipped with an incubator set to 37 °C. Control samples of dead cells were produced by treatment of cells with 0.2% digitonin in PBS for 5 min prior to incubation with dyes.

### 2.9. Statistical analysis

Statistical analysis was performed using Student’s t-test. The results shown are triplicate measurements (mean ± SD, SD = standard deviation) obtained from at least three independent experiments.

## 3. Results

### 3.1. PUR-scaffold structure is independent of the exact formulation

**Figure 2.**
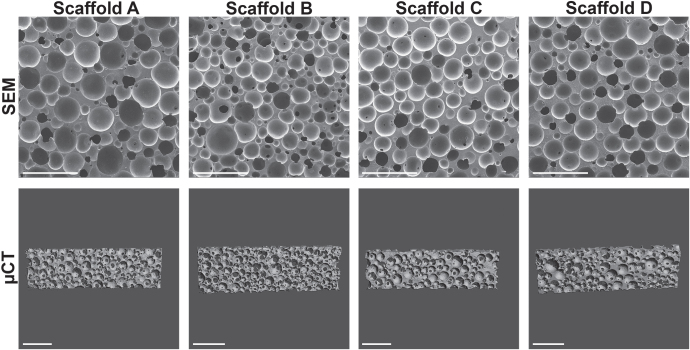
Characteristics of PUR scaffolds. PUR scaffolds A, B, C, and D were imaged by scanning electron microscopy (SEM) or *μ*-computed tomography (*μ*CT) with corresponding measurements shown in table 1(a) (scale bar 1 mm).

To evaluate the structure and homogeneity of the PUR scaffolds, the samples were analyzed by scanning electron microscopy (SEM) and *μ*-computed tomography (*μ*CT) (figure 2). SEM-image analysis revealed similar pore sizes and pore size distributions for all formulations, which was confirmed by the *μ*CT. Structural parameters were computed from the obtained CT scans, showing porosity values of 68–77%, pore thickness of 100–150 *μ*m, and wall thickness of 35–51 *μ*m (table 1(a)). Pore size and wall thickness distributions are displayed in figure s1.

**Table 1.** Characteristics of PUR scaffolds. Data are expressed as mean ± SD (a) porosity values (*n* = 2), (b) compression hardness at RT (*n* = 5–6) and 37 °C/90% relative humidity (r.h.; *n* = 5–6).

		Scaffold A	Scaffold B	Scaffold C	Scaffold D
(a) Porosity (%)		78 ± 2	76 ± 1	73 ± 2	68 ± 1
Pore size (*μ*M)		150 ± 19	114 ± 5	136 ± 12	104 ± 18
Wall thickness (*μ*M)		43 ± 1	36 ± 3	52 ± 1	48 ± 6
(b) Compression hardness (kPa)	RT/dry	73	41	26	38
	37 °C/90% r.h	20	22	15	30

**Figure 3.**
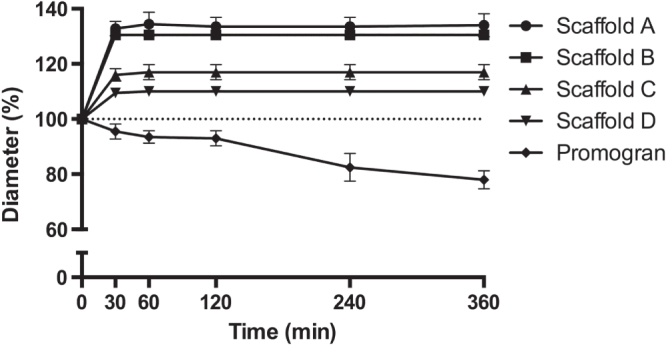
Swelling of PUR scaffolds. PUR scaffolds were immersed in PBS for 30, 60, 120, 240, and 360 min before the diameter was measured (20 mm at *t* = 0; *n* = 2, mean ± SD).

The mechanical properties of the scaffolds were evaluated by compression tests in both the dry and the wet state. Depending on the formulation, different compression hardnesses could be observed. It is interesting that, although scaffold D showed intermediate hardness in the dry state, wetting resulted in only a 19% reduction in hardness. In contrast, the hardness of the other scaffolds was reduced by 43–73% as result of wetting (table 1(b)). When the samples were wetted, their diameters increased by 10% (scaffold D) to 35% (scaffold A) within the first 30 min and remained constant for the rest of the observed period of time (figure 3). The height of the samples tended to increase over this time period. This increase was, however, within the limitations of the measurements (data not shown). Notably, the sample diameter of the reference material Promogran shrank by 22% over the course of 3 h. Furthermore, the thickness of the Promogran greatly decreased (by approximately 15–20%), indicating that its porous structure collapsed upon wetting (data not shown).

### 3.2. PUR-scaffolds display formulation-dependent degradation rates

**Figure 4.**
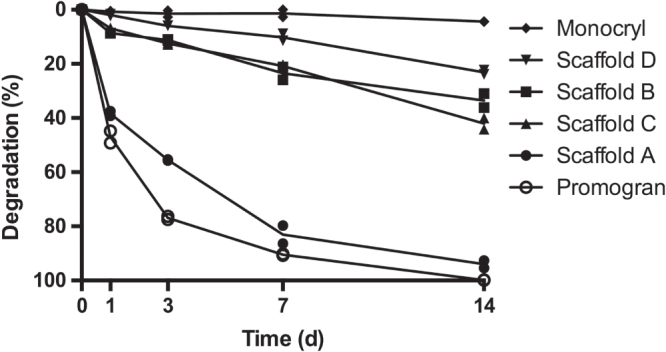
Degradability of PUR scaffolds. PUR scaffolds were submersed in 20% H_2_O_2_ and incubated for 1, 4, 7, and 14 days before the extent of degradation was assessed. Promogran and Monocryl served as fast and non-degrading control materials, respectively. (*n* = 2; each data point represents the mean of triplicate measurements.)

**Figure 5.**
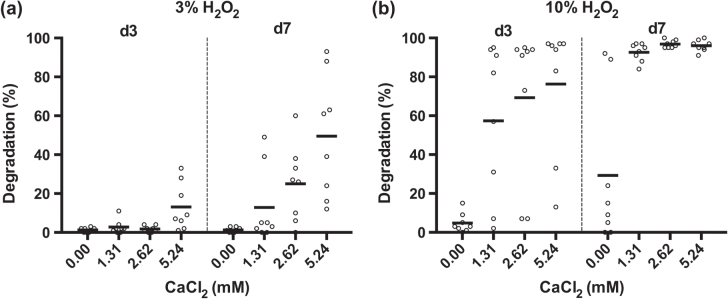
Influence of CaCl_2_ on the degradation rate of PUR scaffold A. PUR scaffold A was submersed in (a) 3% H_2_O_2_ or (b) 10% H_2_O_2_, both supplemented with 0, 1.31, 2.62, or 5.24 mM CaCl_2_ and incubated for 3 and 7 days before the extent of degradation was assessed (*n* = 4, mean of duplicate measurements).

The degradability of the PUR scaffolds was assessed by incubating the samples in 20% H_2_O_2_ for up to two weeks with subsequent measurement of the mass loss. When the PUR scaffolds were incubated in 20% (v/v) H_2_O_2_, scaffold A showed a degradation of 37%, 86%, and 95% by days 1, 7, and 14, respectively (figure 4). Scaffolds B and C, in contrast, showed only little degradation by day 3, with values reaching 36% and 40% by day 14. Sample D displayed an even slower degradation, reaching 10% by day 7 and 22% by day 14. The reference material Promogran showed a degradation pattern similar to that of scaffold A. Like scaffold A, it was completely degraded by day 14. In contrast with the other evaluated materials, the scaffold structure of Promogran had collapsed by day 1. The second reference material, Monocryl, showed almost no degradation after 14 days. Of interest is that the oxidative degradation rate was greatly affected by the presence and concentration of calcium ions (figure 5). In evaluating scaffold A, almost no degradation could be observed with 3% H_2_O_2_ on days 3 and 7. However, the addition of CaCl_2_ increased the degradation rate in a concentration-dependent manner (figure 5(a)). Similar degradation behavior was found when using 10% instead of 3% H_2_O_2_ (figure 5(b)).

### 3.3. Leachable concentrations of PUR scaffolds are not toxic

**Figure 6.**
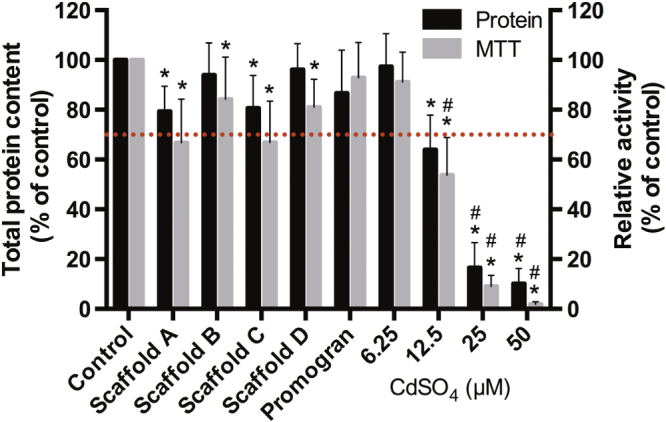
Toxicity of PUR-scaffold extracts. PUR-scaffold extracts obtained by submersing the samples in a medium for 24 h were added to 3T3 mouse fibroblasts for 24 h, and metabolic activity (via MTT) and total protein content were evaluated. A concentration series of CdSO4 served as the negative control. (*n* = 7, mean ± SD; ∗: *p* < 0.05 different from control. #: *p* < 0.05 significant below 70% value).

To evaluate whether non-crosslinked components are released at toxic concentrations from the PUR scaffolds, PUR scaffold extracts were subjected to HPLC-MS before assessment of cytotoxicity by measuring MTT conversion as an index of metabolic activity and total protein content. As only the leachable polyurethane catalyst DMDEE (figure s2) could be detected via HPLC-MS (data not shown) and when exposing 3T3 mouse fibroblasts for 24 h with the 24 h extracts, only a 9–25% reduction in total protein content as well as a 14–34% reduction in metabolic activity could be observed (figure 6). Of importance is that none of the values were significantly below 70% of the control values, which, according the ISO10993-5 standard, is seen as an acceptable reduction threshold. This suggests that none of the materials release acute cytotoxic constituents. Also, the protein and metabolic activity values of the individual scaffolds were not significantly different from one another.

### 3.4. Cell adhesion, but not viability, is dependent on the formulation of the PUR scaffolds

**Figure 7.**
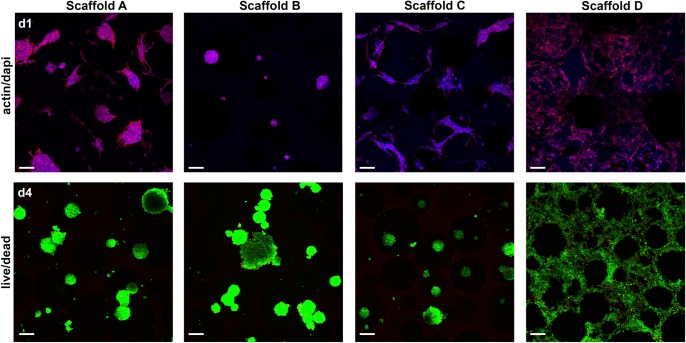
Cell adhesion and viability of scaffold cultivation on PUR scaffolds *in vitro*. Pre-incubated (24 h) PUR scaffolds were seeded with 3 × 10^5^ 3T3 mouse fibroblasts for 24 h (d1) and 96 h (d4) before staining for actin (red) and nuclei (blue) or ethidium homodimer-1 (red) and calcein AM (green), respectively (scale bar 100 *μ*m).

The ability of the cells to adhere to the PUR scaffolds was assessed by cultivating 3T3 mouse fibroblasts for 24 h and 96 h on the samples (figure 7). To visualize the cells on the scaffold, the cells were stained thereafter for the actin cytoskeleton and the nuclei. After 24 h, the cells formed clusters on scaffolds A through C but showed good attachment to and spreading on scaffold D. Similar behavior could be seen after 96 h, with cell clusters on scaffolds A and C, indicating improved attachment and spreading but not reaching the degree of spreading observed on scaffold D.

To evaluate a possible adverse effect of PUR scaffolds on cells contacting the scaffold, a live/dead assay was performed (figure 7). The cell clusters formed on scaffolds A through C displayed bright green staining (living cells) and only a few red-stained dots (dead cells). Similarly, the well-spread cells on scaffold D were mostly bright green with, however, slightly more cells showing red-stained nuclei compared with the other scaffolds. Overall, this clearly shows good viability of cells cultivated on PUR scaffolds.

### 3.5. Cells show good ingrowth into PUR scaffold D

**Figure 8.**
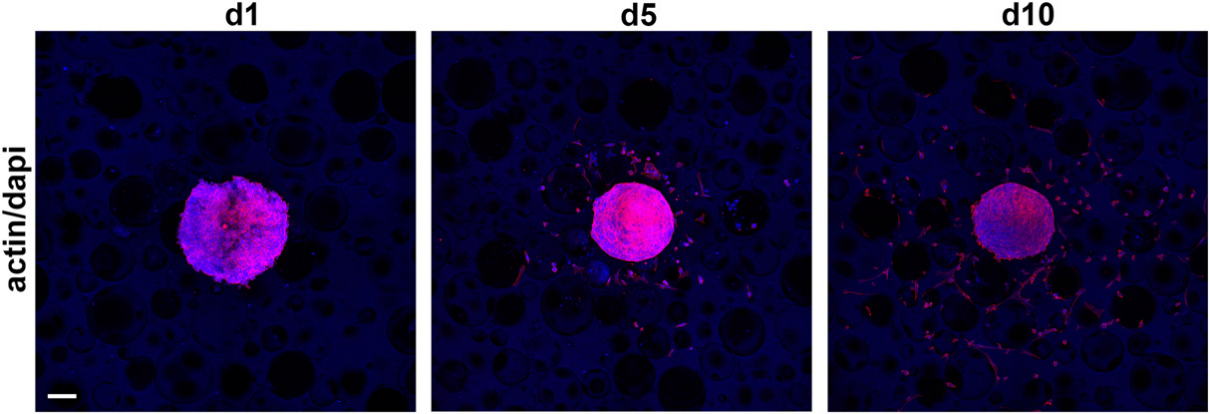
Cell ingrowth into PUR scaffold D from spheroid cultures *in vitro*. Pre-incubated (24 h) PUR scaffolds were seeded with cell spheroids consisting of 1.5 × 10^4^ human dermal fibroblasts (HDFs) for 24 h, 5 days, and 10 days before staining for actin (red) and nuclei (blue, scale bar 100 *μ*m).

With scaffold D allowing very good cell attachment and spreading, the ability of cells to grow into the scaffold was tested by cultivating clustered cell spheroids consisting of 1.5 × 10^4^ human dermal fibroblasts (HDFs) on PUR scaffolds for 24 h, 5 days, and 10 days (figure 8). Thereafter, the actin cytoskeleton and the nuclei were stained to visualize the cells. The cell spheroids showed good attachment after 24 h, and single HDFs grew out into the scaffold by day 5. Robust outgrowth from the spheroid and migration of the cells into the scaffold could be observed after 10 days, with single cells showing good cell attachment and spreading.

## 4. Discussion

In the present study a set of new polyurethane foams as possible scaffold candidates for skin substitutes were characterized.

Based on the premise of an optimal wound dressing, the ideal scaffold should have the following characteristics [26]: (i) three-dimensional and highly porous, with an interconnected pore network for cell/tissue growth and flow transport of nutrients and metabolic waste; (ii) easily able to be processed to form a variety of shapes and sizes; (iii) suitable surface chemistry for cell attachment, proliferation, and differentiation without eliciting an inflammatory or allergic response; (iv) biodegradable or bioresorbable, with a controllable degradation and resorption rate to match cell/tissue growth *in vitro* and/or *in vivo*; (v) mechanical properties to match those of the tissues at the site of implantation. Furthermore, protection from bacterial invasion is seen as additional ideal characteristic [8].

Our currently developed set of materials exhibited characteristics which, depending on the composition, meet all or nearly all of these requirements:

(i) and (ii) **Scaffold morphology and processing.** The evaluated scaffolds are soft wound surface adaptable and can be simply cut with knives or scissors in any desired shape before application. Injectable PUR scaffolds offer more flexibility in terms of tissue repair [27, 28] and better adaptability to the wound bed in the case of the skin [29]. However, it can be expected that access to the foam-shaped scaffolds described herein from the tissue side is improved due to the high porosity with defined partially interconnected pores, which are shape stable for several days. Of importance is that during production, pore size and pore size distribution can be influenced by altering the processing parameters, e.g., pressure (data not shown), which allows for even further optimization of the scaffold structure. Overall, this proves that the PUR material can be easily processed as three dimensional and highly porous with an interconnected pore network for cell/tissue growth and flow transport of nutrients and metabolic waste.

(iii) **Cell attachment.** The fibroblast cells displayed good attachment to the surface of the materials, which was especially apparent in the case of scaffold D, where excellent adhesion of a single cell layer could be observed. Furthermore, simulating tissue ingrowth with clustered cell spheroids showed that cells were able to colonize the obviously nontoxic materials. The cyto-compatibility of the materials was further demonstrated by a live/dead assay showing excellent cell viability. This is in good agreement with previous studies of biodegradable PUR scaffolds showing good cyto-compatibility [30, 31]. Evaluation of PUR scaffold extracts showed only minor, insignificant reduction of cell metabolic activity and protein content—observations similar to previous studies [31, 32]. Thus, our data gives clear evidence that the PUR material is not toxic and exhibits suitable surface chemistry for cell attachment, proliferation, and differentiation.

(iv) **Degradation.** It was found that the degradation velocity of the scaffolds was dependent on various factors, i.e., the degradation method used, the composition of the degradation medium, and the composition of the scaffold material. In PBS and water, no degradation could be observed by day 28 (data not shown). This is in good agreement with previous studies of biodegradable polyurethane scaffolds, which showed only little to no degradation after four weeks in PBS [17, 20, 30, 33]. However, when using hydrogen peroxide solutions to test for oxidative degradation of the PUR scaffolds, the materials showed that degradation rates very much depended on the exact formulation, being tunable from as little at 20% to complete degradation within 14 days. Of interest is that the degradation rates were greatly influenced by the presence and concentration of the divalent metal cation calcium. Incubated at a physiological concentration of 3% H_2_O_2_, the PUR scaffolds showed no degradation by day 7. However, when adding 5.24 mM CaCl_2_ to the solution, a mass loss of 50% could be observed under otherwise identical conditions. It is interesting that accelerated degradation could not be observed upon supplementation with either magnesium or iron (data not shown). Such an auto-oxidative effect of specific metals has long been known [34] and has been exploited to study the biostability of PUR materials [35]. The positive influence of calcium on PUR scaffold degradation is especially interesting since, dependent on the skin layer, a gradient from 0.5 to 1.4 mM of calcium can be found in mammalian skin [36]. Furthermore, these levels have been shown to increase up to fivefold during skin wound healing [37], which may facilitate PUR scaffold degradation *in vivo*. Preliminary results in rats support this hypothesis, with complete degradation being observed after several days to a few weeks (data not shown). Chronic wounds, on the other hand, can have a deregulated balance of trace metal concentrations [38], and thus, availability of calcium for an accelerated degradation of the PUR scaffold may be limited in such an environment. This can probably be compensated by wetting the PUR scaffold using a calcium-containing Ringer solution with 2.2 mM Ca^2+^ prior to or just after application, which has the further advantage that calcium has a recognized positive effect on wound healing [36]. Therefore, our data clearly show that the PUR material is biodegradable or bioresorbable, with a controllable degradation and resorption rate to match cell/tissue growth *in vitro* and, as preliminary data show, also *in vivo* (publication in preparation).

(v) **Mechanical properties.** A further interesting characteristic of the tested materials is their swelling property upon wetting. Thereby, good adaptability and firm contact with the wound bed can be achieved. This is in contrast with the reference material Promogran, which, while demonstrating good performance *in vivo* [39], collapsed upon wetting. Swelling is, however, also beneficial for the wound healing process, as it builds a small amount of pressure on wound boundaries. This may increase the contact with this tissue and decrease wound contraction and, as a result, stimulate cell ingrowth and likely wound healing. Furthermore, although the tested PUR scaffolds are flexible, they are form-stable for days, and it can thus be expected that they, in contrast with, e.g., collagen matrices, will withstand the contractile forces generated by cells. This resilience might give the materials tested herein a critical advantage over less form-stable scaffolds, as it will likely hamper the formation of myofibroblasts as recently shown in *in vivo* experiments using these scaffolds (publication in preparation), which facilitate scar formation during wound healing [40]. These data provide evidence that the PUR material has, besides being biocompatible, optimal mechanical properties matching those needed for the skin to support wound healing.

(vi) **Infections.** All chronic wounds are in a persistent pro-inflammatory state that is multifactorial related to local tissue hypoxia, necrosis, and a heavy bacterial burden that delays or hinders healing by impaired cell migration and reduced fibroblast proliferation and collagen synthesis [41]. However, equipping or soaking the PUR scaffolds from this study with antimicrobial substances (e.g., ionic silver, molecular iodine, activated carbon, sulfonamide, or polyhexamethyl biguanide) that provide continuous or sustained release of these agents may potentially alleviate this problem.

## 5. Conclusions

In conclusion, the PUR scaffolds tested present a wound dressing concept that fulfills the key criteria for an optimal scaffold for tissue engineering and thereby provide a material that has the potential to meet the requirements for an ideal wound dressing. This has been supported by very promising preliminary results in rats but has yet to be fully evaluated before translation into clinics.
